# Beneficial effects of nicotinamide on hypertensive mice with impaired endothelial nitric oxide function

**Published:** 2020

**Authors:** Phillip K Huynh, Jen Wilder, Sylvia Hiller, John Hagaman, Nobuyuki Takahashi, Nobuyo Maeda-Smithies, Feng Li

**Affiliations:** 1 Department of Pathology and Laboratory Medicine, The University of North Carolina, Chapel Hill, NC 27599, USA; 2 Division of Clinical Pharmacology and Therapeutics, Tohoku University Graduate School of Pharmaceutical Sciences, Sendai, Japan

**Keywords:** Nicotinamide, Mice, L-NAME, Blood pressure, Endothelial nitric oxide synthase

## Abstract

Nicotinamide (Nam, amide form of niacin acid or nicotinate), a precursor for nicotinamide adenine dinucleotide (NAD+), is important for normal physiological function of organisms. Nam also suppresses mobilization of Ca^2+^ from sarcoplasmic reticulum into cytoplasm through inhibiting ADP-ribose cyclase. Previously, we have demonstrated that a pharmacological dose of Nam normalizes maternal blood pressure in mouse models of preeclampsia, a pregnancy related hypertensive disorder. We hypothesized that Nam could decrease blood pressure in hypertensive conditions unrelated to pregnancy. Nam at a dose of 500 mg/kg/day was given to wild type (WT) mice treated with L-NAME, endothelial nitric oxide synthase (eNOS)-null and renin transgenic (Renin-Tg) mice via drinking water. Blood pressure was measured by tail-cuff at different stages of treatment. The function and structure of kidneys of WT mice with L-NAME were determined at the end of the study. The gene expression of markers of inflammation and fibrosis in the kidneys of WT mice with L-NAME was also measured. Nam effectively prevented increase in blood pressure in L-NAME treated mice and decreased elevated blood pressure in eNOS-null mice. However, it did not alter high blood pressure in Renin-Tg mice. Nam prevented increase in urinary albumin excretion and collagen deposit in kidneys of WT mice treated with L-NAME. In addition, Nam significantly decreased the mRNA levels of the markers of inflammation and fibrosis in the kidneys of WT mice treated with L-NAME. Nam may execute beneficial effects on hypertensive conditions associated with eNOS dysfunction via suppressing inflammation. Because Nam is generally regarded as safe in humans, it merits further evaluation for the tailored treatment for the subgroup of hypertensive cases associated with impaired eNOS system.

## Introduction

Nicotinamide (Nam, amide form of niacin acid or nicotinate) along with niacin acid and nicotinamide riboside are components of an essential vitamin (vitamin B3). These compounds and tryptophan are precursors for nicotinamide adenine dinucleotide (NAD_+_) and nicotinamide adenine dinucleotide phosphate (NADP) in most living organisms [1]. The classical function of NAD_+_/NADP is to act as a coenzyme for hydride-transfer enzymes, which is central to metabolism, including energy production and synthesis of fatty acids, cholesterol, and steroids. Insufficiency of NAD_+_/NADP resulting from deficiency of its precursors can lead to clinical manifestation including fatigue, pigmented skin rash, and oral ulcerations [2]. In severe deficiency, patients develop pellagra which is characterized by cutaneous rashes and dermatitis, diarrhea, and dementia [1]. Elvehjem demonstrated that Nam and niacin acid had anti-pellagragenic effects in 1937 [3]. The recommended daily allowance of vitamin B3 is13–16 mg per day.

Besides its function as coenzyme, NAD_+_ also is a substrate of NAD_+_-consuming enzymes that cleave the N-glycosidic bond between the Nam moiety and the ADP-ribose moiety. One or more ADP-ribose moieties are transferred to certain proteins which are reversibly modified in order to form signaling compounds from NAD_+_ and NADP [1,4]. For example, ADP-ribosyl cyclase catalyzes the conversion of NAD_+_ to cyclic ADP-ribose (cADPR) and Nam. While cADPR mobilizes intracellular Ca^2+^ from sarcoplasmic reticulum via actions on ryanodine receptors [5], Nam inhibits ADP-ribosyl cyclase [6] which can be activated by the endothlin-1 (ET-1)/endothelin receptor system and other factors [7]. Recently, we have reported that Nam decreases maternal blood pressure and improves maternal kidney function and structures in mouse models of preeclampsia via inhibiting ADP-ribosyl cyclase [8]. Preeclampsia is a hypertensive disorder that occurs in pregnant women [9]. The molecular mechanisms of this condition is largely unknown and there is no effective therapeutic strategy available.

In the current study, we hypothesized that Nam may also lower blood pressure in broader spectrum of hypertensive conditions. Therefore, we tested the blood pressure lowering effects of Nam on hypertension caused by inhibiting endothelial nitric oxide synthase (eNOS). eNOS/nitric oxide (NO) plays an important role in blood pressure regulation, as evidenced by genetic deletion of *Nos3* (encoding eNOS) gene leading to hypertension in mice [10,11]. In addition, pharmacological inhibition of eNOS leads to hypertension as well. Although N^G^-nitro-L-arginine methyl ester (L-NAME) is a potent non-selective inhibitor of all three isoforms of NOS [eNOS, inducible nitric oxide synthase (iNOS) and neuronal nitric oxide synthase (nMOS)], it has been widely used to induce hypertension due to inhibiting eNOS [12–14]. Our results demonstrate that Nam prevents against increase in blood pressure and kidney injury in L-NAME treated wild type (WT) mice, and decreases elevated blood pressure in eNOS-null mice. In addition, Nam suppresses inflammation and fibrosis in kidneys of WT mice treated with L-NAME

## Materials and Methods

### Mice

Wild type male (WT) mice (C57BL/6J), Renin-Tg [129S/ SvEv-Tg(Alb1-Ren)2Unc/CofJ, JAX: 007853] male mice and eNOS-null female and male mice (on the C57BL/6J background) [15] at age of 12–16 weeks were housed in standard cages on a 12h light/dark cycle and were allowed free access to food and water. All experiments were carried out in accordance with the National Institutes of Health guideline for use and care of experimental animals, as approved by the IACUC of the University of North Carolina at Chapel Hill.

### WT mice with L-NAME and/or Nam treatment

WT mice were randomly enrolled into four groups: 1) control: mice received only vehicle (water), 2) Nam: mice were administered with Nam (#72340, Sigma) at a dose of 500 mg/kg/day in drinking water (0.3% weight/volume) [8], 3) L-NAME : mice were treated with L-NAME (#483125, Sigma) at dose of 50 mg/kg/day in drinking water (0.03% weight/volume) [14], 4) L-NAME + Nam: mice were treated with both L-NAME and Nam via drinking water at dose described in 2) and 3) respectively. After 2 months treatment, mice were euthanized, and body fluids and tissues were collected for analysis.

### Renin Tg and eNOS-null mice with Nam treatment

Renin Tg and eNOS-null mice were randomly enrolled into either control (vehicle: water) or Nam (same dose as described above) groups. Blood pressure was measured before and after 1-month of treatment.

### Blood pressure measurement

The blood pressure of mice was determined via transmission photoplethysmography and an occlusion tail cuff (BP-2000 Blood Pressure Analysis System; Visitech Systems, NC) as described previously [16].

### Urinary albumin

Urine was collected by massaging the bladder at one time, and urinary albumin concentration and creatinine were determined using commercially available kits (Exocell Inc., Philadephia, PA) as described previously [17].

### Morphological examination

Kidney tissues were fixed with 4% paraformaldehyde, paraffin sectioned (5 μm), and stained with hematoxylin and eosin (H&E), or with Masson’s Trichrome [17].

### Quantitative RT-PCR

Total RNA from tissues was extracted using Trizol (Life Technologies, St. Paul, MN) following the manufacturer’s instruction. mRNA was quantified with TaqMan real-time quantitative RT-PCR (7500 real time PCR system, Applied Biosystems, Foster City, CA) by using the one-step RT-PCR Kit (Bio Rad, Hercules, CA) with *Hprt* as the reference gene in each reaction [18].

### Statistical analysis

Data are presented as mean ± SEM. Multifactorial ANOVA test was used to assess statistical significance with the program JMP 14.0 (SAS Institute Inc. Cary, NC). Post hoc analyses were done using the Tukey–Kramer Honest Significant Difference test.

## Results

### Nam prevents increase in blood pressure in wild type (WT) mice chronically treated with L-NAME

**Figure 1 :**
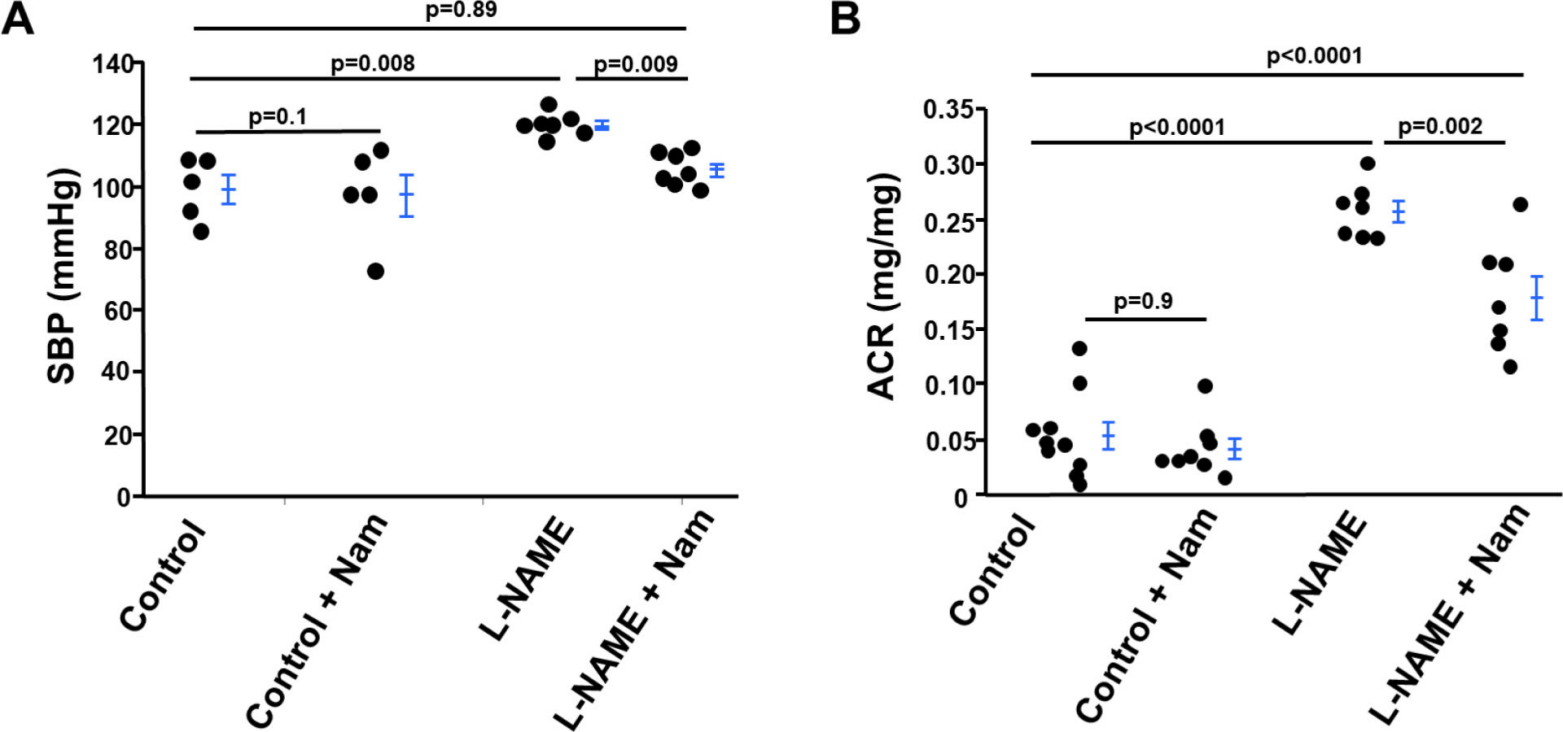
Nicotinamide (Nam) protects against increase in blood pressure and urinary albumin excretion in wild type (WT) mice treated with L-NAME. **(A)** Systolic blood pressure (SBP) of four groups of mice was measured after 2-months of treatment. n≥5. **(B)** Urinary albumin/ creatinine ratio (ACR) was determined after 2-months of treatment n≥7.

We tested the effect of Nam on hypertension induced by L-NAME treatment. After treatment with L-NAME for 2 months at a dose of 50 mg/kg/day, the blood pressure of WT mice increased approximately 20 mmHg, consistent with previous reports [14], and Nam restored the blood pressure to normal control levels (Figure 1). Nam alone had no effect on blood pressure of WT control mice without L-NAME.

Neither Nam nor L-NAME had effect on body weight, and heart and kidney weight in WT mice under the treatment regimen of this study (Table 1).

### Nam improves kidney function in wild type (WT) mice chronically treated with L-NAME

After treatment with L-NAME for 2 months at a dose of 50 mg/ kg/day, kidney function was damaged as indicated by the increased urinary protein excretion as measured by albumin/creatine ratio (ACR), while Nam significantly dampened this induction. Nam had no effect on ACR in WT control mice (Figure 1B).

### Nam suppresses inflammation in kidneys of wild type (WT) mice induced by L-NAME

Our previous study [8] and pilot data of the current study have not shown any effect of Nam on WT control mice, therefore, we only investigated the difference between mice treated with L-NAME and mice treated with L-NAME plus Nam.

**Figure 2:**
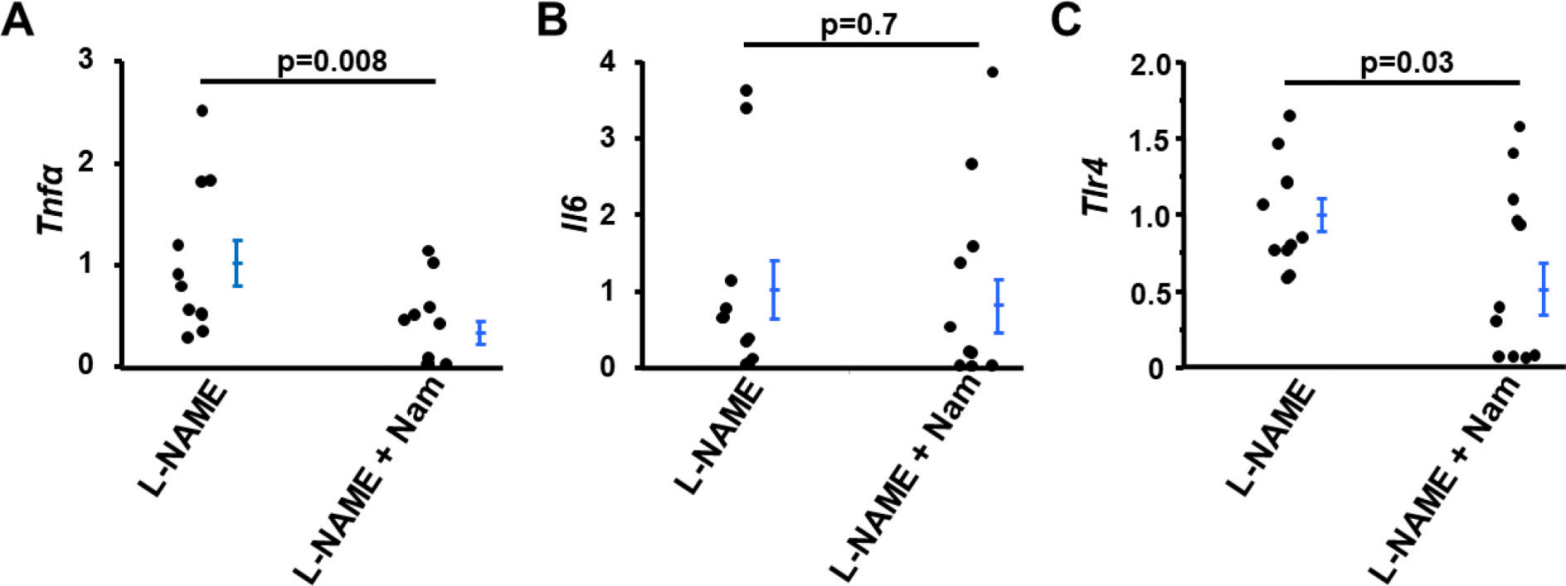
Nicotinamide (Nam) co-treatment suppressed the inflammatory gene expression of Tnfα and Tlr4 but not Il6 in kidneys of WT mice treated with L-NAME. mRNA levels of *Tnfα* **(A)**, *Il6* **(B)** and *Tlr4* **(C)**. n≥10.

L-NAME induces tumor necrosis factor-α (TNFα) expression in kidneys and other tissues [13,14] and Nam has been shown to inhibit TNFα signaling induced by lipopolysaccharide (LPS) [19]. We, therefore, examined the effect of co-treatment with Nam on the expression of the *Tnf*α gene in the kidneys of mice treated with L-NAME. As shown in Figure 2A, mRNA levels of *Tnf*α in kidneys of mice co-treated with L-NAME and Nam was 30% that in kidneys of mice treated with L-NAME only (p<0.008). In contrast, Nam co-treatment did not affect the mRNA levels of *Il6* (encoding Interleukin 6, IL6) in the kidneys of WT mice treated with L-NAME (Figure 2B), consistent with Fukuzawa et al. report that Nam had no significant effect on IL6 production induced by LPS [19].

Toll-like receptor4 (TLR4) is an important contributor of the innate immune system and is involved in the pathological changes induced by L-NAME [14,20]. Therefore, we examined the effect of Nam on the mRNA level of *Tlr4.* Like its effect on mRNA level of *Tnf*α, the mRNA levels of *Tlr4* in kidneys of WT mice co-treated with Nam and L-NAME were markedly lower than that of the L-NAME only treatment group by 50% (p=0.03, Figure 2C).

### Nam suppresses fibrosis in kidneys of WT mice induced by L-NAME

**Figure 3:**
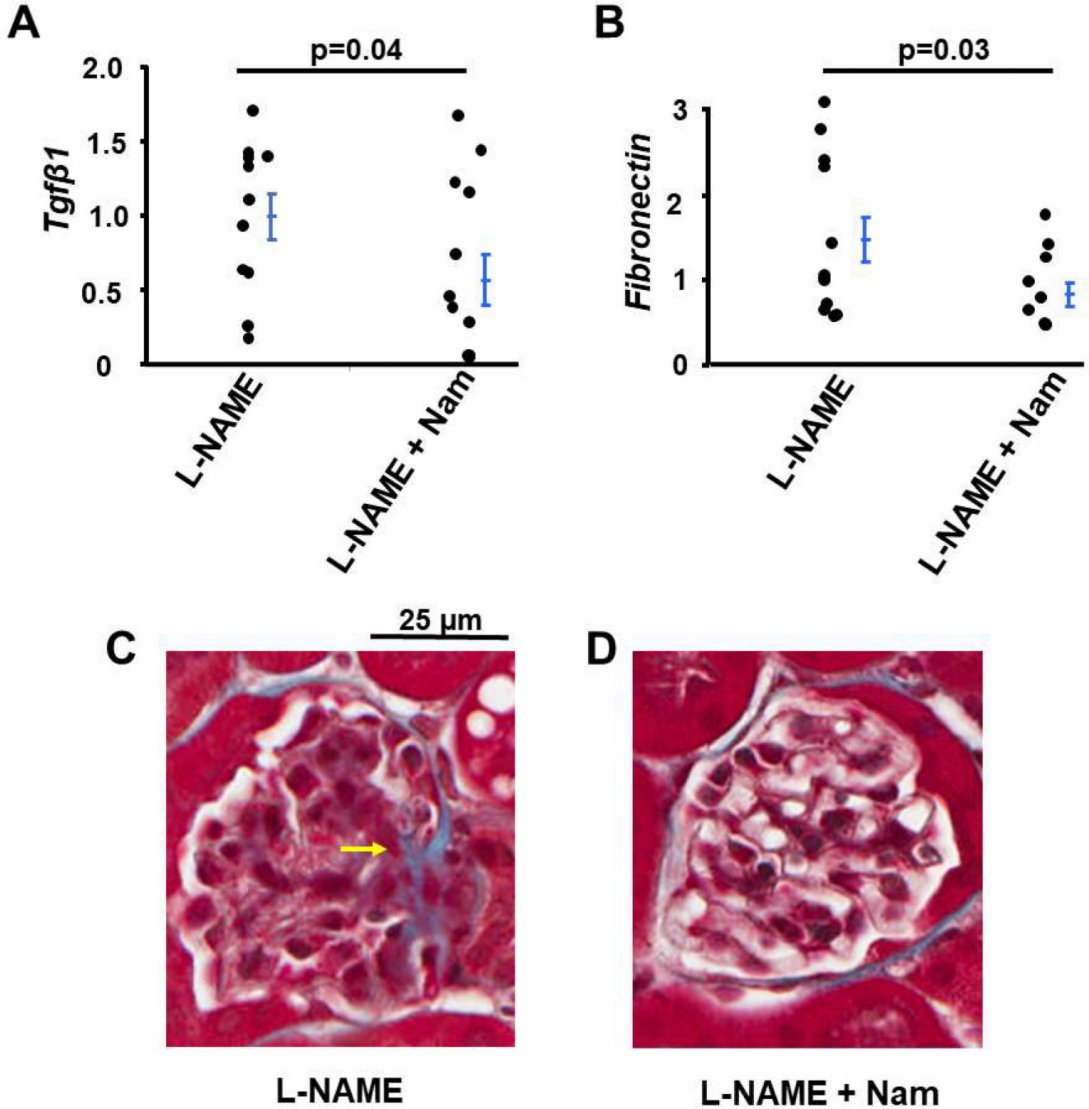
Nicotinamide (Nam) co-treatment suppressed the kidney fibrosis in WT mice treated with L-NAME. mRNA levels of markers of fibrosis genes **(A)** *Tgfβ1*, **(B)** *Fibronectin.*n≥10 **(C)** Representative Masson’s Trichrome stain of kidneys in the two different groups of mice. Yellow arrow: fibrosis in a glomerulus which was not present in kidneys of mice treated with L-NAME plus Nam.

L-NAME treatment leads to kidney fibrosis [13] and TGFβ1 is thought to play a role in the pathogenesis of fibrosis caused by L-NAME [21]. We examined the effect of Nam on kidney fibrosis of mice co-treated with L-NAME. The mRNA levels of *Tgfβ1* and *Fibronectin* in the kidneys of WT mice co-treated with Nam and L-NAME were about 50% of those treated with L-NAME only (Figures 3A and 3B). In addition, Nam decreased collagen deposition detected by Masson’s Trichrome staining in kidneys of WT mice with L-NAME treatment (Figures 3C and 3D).

### Nam decreases blood pressure in eNOS-null mice

**Figure 4:**
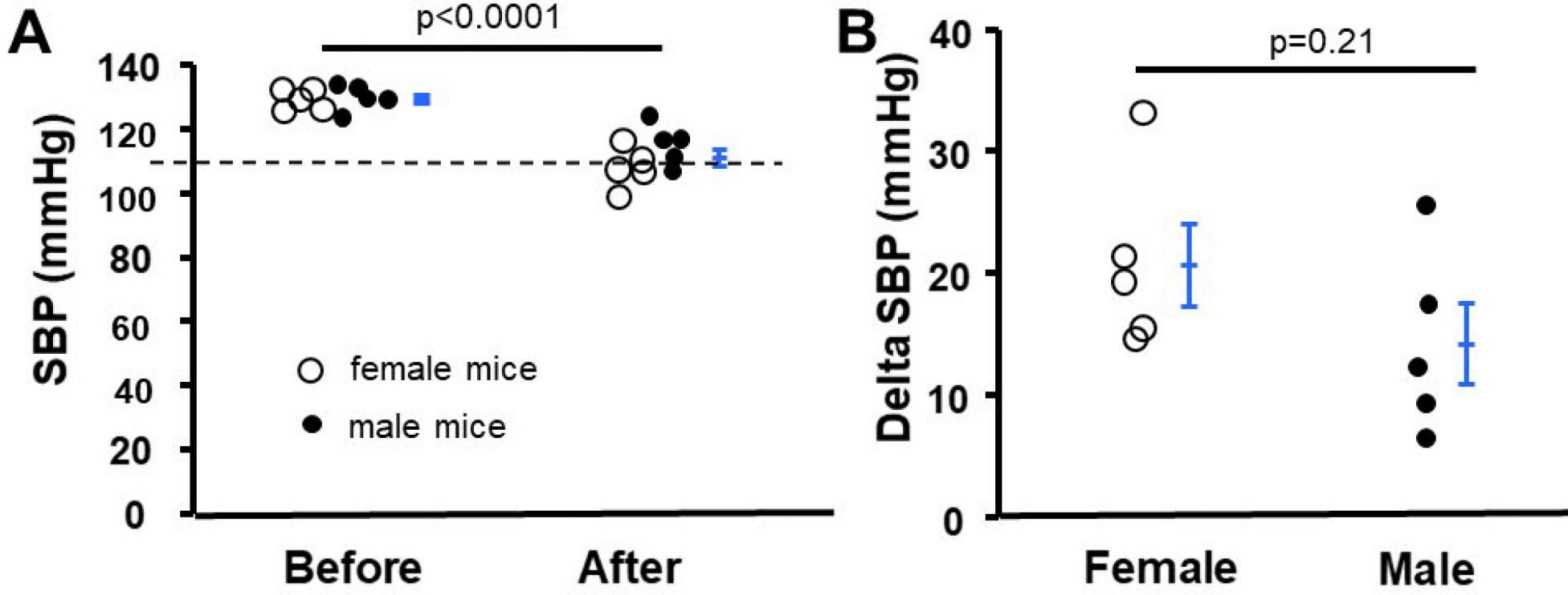
Nicotinamide (Nam) decreased elevated blood pressure in eNOS-null mice. **(A)** Systolic blood pressure (SBP) was measured by tail-cuff before and 1-month after treatment. n=5 female and 5 male mice. Broken line indicates the value of SBP of WT mice at the same age. **(B)** The difference of SBP before and 1-month after Nam treatment in male and female mice. n=5.

Genetically lacking eNOS results in hypertension in mice [10,11]. We tested the blood pressure lowering effect of Nam on this hypertensive mouse model. Before Nam treatment, the systolic blood pressure (SBP) of eNOS-null mice was 129.2 ± 1.1 mmHg, the SBP significantly decreased to 111.3 ± 2.3 mmHg after 1-month treatment (p<0.0001, Figure 4A). Nam effectively decreased blood pressure in both female and male eNOS-null mice, although female mice tended to decrease more (p=0.21, Figure 4B). Because eNOS-null mice do not develop obvious kidney problems [15], we did not examine the kidney function and structure in these mice.

Taken together, we demonstrated that Nam had antihypertensive effects in mice with impaired eNOS function.

### Nam did not alter blood pressure in Rein-Tg mice and did not alter the expression of *Renin* gene in kidneys of wild type (WT) mice treated with L-NAME

**Figure 5:**
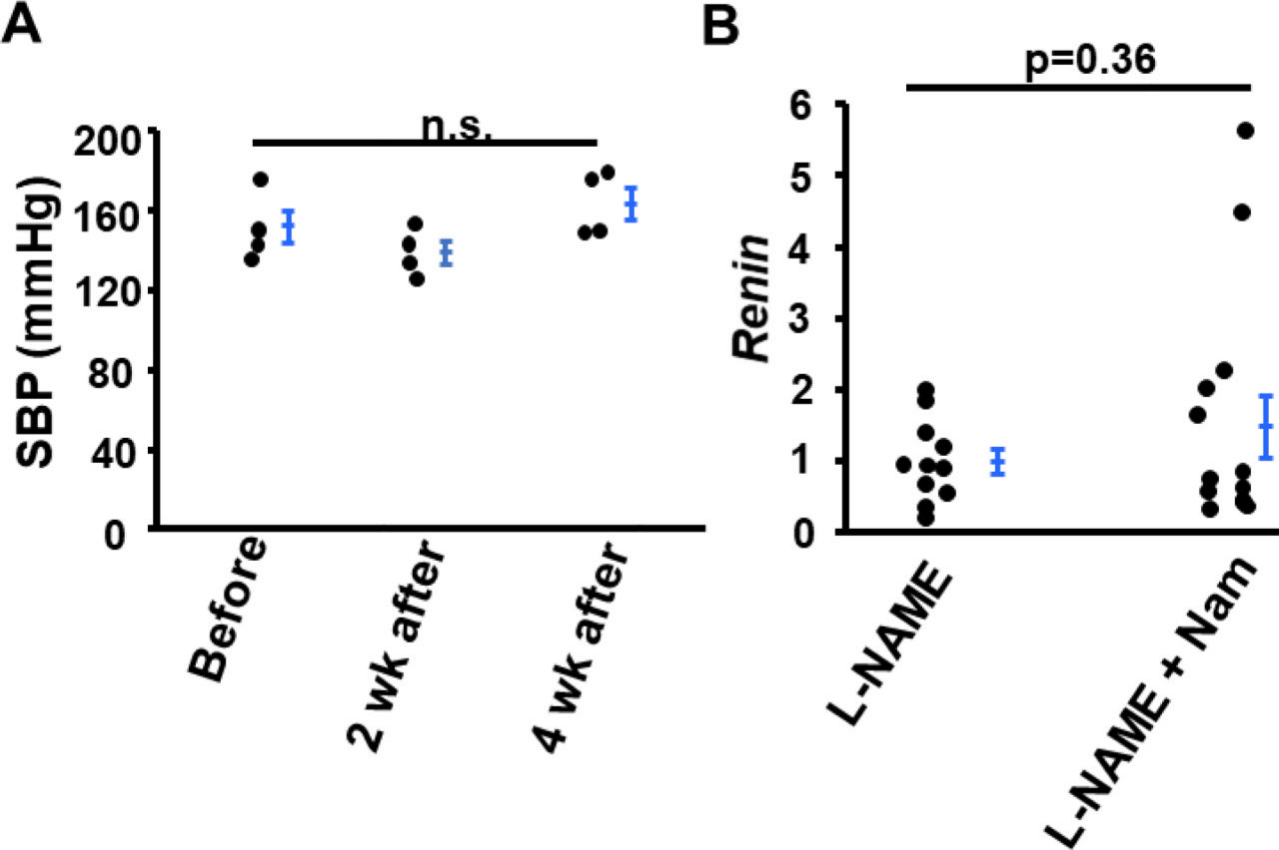
Nicotinamide (Nam) did not alter blood pressure in Renin Tg mice and mRNA level of Renin in kidneys of WT mice treated with L-NAME. **(A)** Systolic blood pressure (SBP) was measured before, 2-weeks and 4-weeks after Nam treatment. n.s.: not significant. n=4. **(B)** mRNA level of *Renin*. n≥10.

We next tested whether Nam decreased blood pressure in mice in which hypertension was not resulting from eNOS dysfunction. Therefore, we treated Renin-Tg mice with Nam for 4 weeks and blood pressure was measured before, 2 and 4 weeks after treatment. The Renin-Tg mice express renin ectopically at a constant high level in the liver which leads to elevated plasma levels of prorenin and active renin. The transgenic mice display high blood pressure, and kidney damage [22]. Before Nam treatment, the SBP of Renin-Tg mice was 152.4 ± 8.0 mmHg. At 2 weeks and 4 weeks after treatment, the SBP was 139.2 ± 5.9 and 163.7 ± 8.0 mmHg, respectively (Figure 5A). Nam did not decrease elevated blood pressure in mice caused by over-expressing renin [22].

Because the renin-angiotensin system (RAS) is involved in L-NAME induced kidney lesions, we determined the mRNA level of Renin in the kidneys. Nam had no effect on the gene expression (Figure 5B).

## Discussion

In the current study, we demonstrated that Nam effectively decreased blood pressure in mice whose blood pressure were above normal due to pharmacological inhibition of eNOS with L-NAME, or due to genetic lack of *Nos3*. Co-treatment with Nam prevented the decline of kidney function in WT mice treated with L-NAME, as judged by lower urinary albumin excretion. In addition, Nam suppressed kidney inflammation and fibrosis induced by L-NAME.

Nam is generally considered safe by the Food and Drug Administration (FDA), and daily doses of over 3 g are generally well tolerated [23]. The dose used in our study with mice translates to approximately 2.5 g/day in a person whose body weight is 60kg [24]. Nam appears to be a well-tolerated medication with broad applications. For example, Nam has been used to treat acne vulgaris and other skin problems for more than 50 years [25,26]. Although the precise mechanism is not very clear, inhibition of proinflammatory cytokine pathways has been proposed as the underlying mechanism of its beneficial effects in the skin conditions [25]. Because Nam is known to inhibit poly-ADP-ribose-polymerase-1 (PARP-1) and regulate DNA repair, it has potential as a prophylaxis against nonmelanoma skin cancer [27,28]. In other diseases, there are clinical trials to test the role of Nam in Alzheimer’s Disease (NCT00580931) and preeclampsia (NCT03419364). More recently, beneficial effects of Nam have been explored in animals with aims to treat human diseases in the future such as age-related macular degeneration [29] and glaucoma [30].

Hypertension is a worldwide health challenge as result of its high prevalence and concomitant risks for stroke and cardiovascular disease. Numerous genetic and environmental factors are involved in pathogenesis and progression of hypertension [31]. In human studies, *NOS3* (encoding eNOS) polymorphisms are associated with hypertension [32–35]. However, there is no tailored therapeutic regiment for hypertension associated with impaired eNOS/NO. In this study, we show that Nam effectively decreased blood pressure in hypertensive mice with impaired eNOS function genetically or pharmacologically without detrimental effects. In contrast, our experiments showed that Nam had no BP-lowering effects in severe hypertension caused by an unregulated overproduction of renin in mice. Taken together, our data suggest that this economic well-tolerated small molecule has tremendous potential as a treatment for the hypertensive condition related to eNOS dysfunction.

Both Inflammation and innate immunity play a role in the development of hypertension [20,36,37]. In humans, eNOS polymorphisms associated with hypertension also modulates the inflammatory response [38]. TLR4 signaling modulates blood pressure in L-NAME-induced hypertension, and Sollinger et al reported that *Tlr4*-null mice are protected against blood pressure increases by L-NAME [14]. Nam has been shown to possess anti-inflammation properties [19,39]. Our current data show that co-treatment with Nam suppressed the mRNA levels of *Tn*α and *Tlr4* in kidneys of mice induced by L-NAME. Thus, the anti-inflammation could be one of the mechanisms by which Nam decreases blood pressure.

In our previous work we have shown that Nam decreases maternal blood pressure through inhibiting ADP-ribose cyclase in preeclamptic mice. This could also be a mechanism by which Nam moderates the development of hypertension in mice lacking eNOS. Further study is needed to elucidate it [8].

Some reagents that decrease the elevated blood pressure induced by L-NAME are thought to work through preserving eNOS/ NO [40]. However, our current study shows that Nam effectively decreases blood pressure in eNOS-null mice, and the mRNA level of *Nos3* is not increased in the kidneys of WT mice treated with L-NAME and Nam (1.00 ± 030 in kidneys with L-NAME vs. 0.28 ± 0.08 in kidneys with L-NAME + Nam, p=0.02), suggesting that Nam decreases blood pressure in an eNOS/NO-independent manner.

L-NAME causes proteinuria and kidney fibrosis [13] and the mechanisms of the chronic pathological changes are more complex than simple inhibition of endothelial NO synthesis. Upregulation of RAS is thought to play an important role in fibrosis of tissues including the heart [41] and kidney [13]. In the current study, we observed that Nam partially corrected the urinary albumin excretion but had no effect on the mRNA level of *Renin* in the kidneys of WT mice treated with L-NAME. These data suggest that Nam could resolve the kidney problems associated with eNOS dysfunction but have no effects on pathological changes induced by uncontrolled upregulation of RAS.

**Figure 6:**
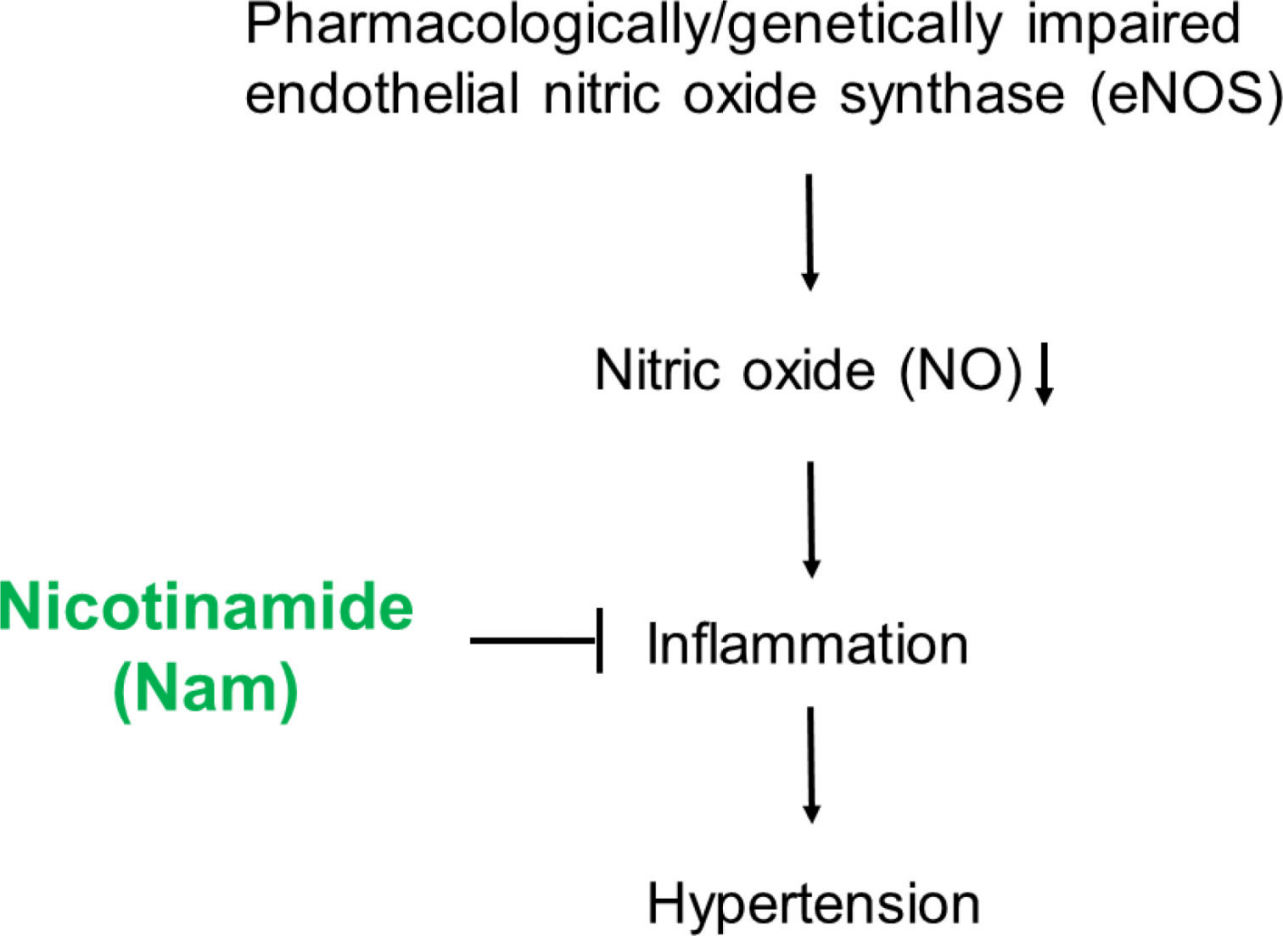
Schematic illustration of the potential mechanism by which nicotinamide (Nam) executes its beneficial effect.

In summary, we have demonstrated that a pharmacological dose of Nam normalizes blood pressure in mice with impaired eNOS function either pharmacologically or genetically, and has partial benefits on kidney problems induced by L-NAME. Because Nam is generally regarded as safe in humans, it merits further evaluation as a treatment of human hypertension associated with eNOS dysfunction (Figure 6).

## Figures and Tables

**Table 1:** Characteristics of different groups of mice.

	Control	Control+Nam	L-NAME	L-NAME+Nam
Body weight (g)	32.4 ± 0.8	33.5 ± 0.8	32.4 ± 1.1	34.6 ± 0.8
Kidney weight (g)	0.198 ± 0.01	0.194 ± 0.004	0.178 ± 0.01	0.199 ± 0.007
Heart weight (g)	0.172 ± 0.002	0.168 ± 0.01	0.170 ± 0.008	0.176 ± 0.0061

Mice were randomly enrolled into four different groups as described in “Materials and Method” section. Two months after treatment, mice were euthanized and plasma and kidneys were collected for the analysis. n≥5.
